# Factors Influencing the Low-Temperature Properties of Styrene-Butadiene-Styrene Modified Asphalt Based on Orthogonal Tests

**DOI:** 10.3390/polym15010052

**Published:** 2022-12-23

**Authors:** Suhua Chen, Enwei Jin, Gang Xu, Shangzhi Zhuo, Xianhua Chen

**Affiliations:** 1 School of Transportation, Southeast University, 2 Sipailou, Nanjing 210096, China; 2 East China Branch of China Railway Bridge Survey and Design Institute Group Co., Ltd., Nanjing 210003, China

**Keywords:** styrene-butadiene-styrene modified asphalt, low-temperature performance, orthogonal test, Glover-Rowe (G-R) parameter

## Abstract

Styrene-butadiene-styrene (SBS) is widely used in asphalt modification to obtain superior high-temperature performance. Nevertheless, studies on the low-temperature properties of SBS-modified asphalt are not satisfactory. Orthogonal tests are valid in analysing the results. In this paper, the main factors (SBS content, sulfur content, and the addition of rubber processing oil) for improving the low-temperature performance of SBS-modified asphalt were analyzed base on the orthogonal tests. Firstly, the frequency sweep test, bending beam rheometer (BBR) test, and force-ductility test were conducted to evaluate the low-temperature properties of SBS-modified asphalt. Investigation of low-temperature parameters obtained through these tests was conducted base on the orthogonal analysis method. The G-R parameter was abandoned in the analysis of the orthogonal method for the result that the increase of SBS content was negative to the low-temperature properties by the Glover-Rowe (G-R) parameter, which were contrary to the results of BBR and force-ductility tests. Moreover, the other parameters (Δ*T_c_* and toughness) sorted according to the orthogonal analysis method indicated the effect on low-temperature performance of the SBS-modified asphalt as SBS content > rubber processing oil > sulfur. As shown above that both SBS and rubber processing oil play a critical role in improving the low-temperature properties of SBS-modified asphalt, for SBS could resist the generation and subsequent propagation of cracks while the rubber processing oil could supplement the maltene loss.

## 1. Introduction

The rapid development of expressways and the increasing traffic loads have prompted styrene-butadiene-styrene (SBS) modified asphalt to reduce the temperature susceptibility, increase the cohesion, and modify the rheological characteristics, which can alleviate the occurrence of early failure of asphalt pavement [1,2,3,4]. Although a considerable amount of research has focused on the SBS-modified asphalt, most of these concentrated on the high temperature properties and performances before and after aging. As the temperature cracks are closely related to the low-temperature cracking resistance of asphalt binders, the low-temperature properties of SBS-modified asphalt have to be comprehensively investigated [5]. Maltenes, which are composed of saturates and aromatics in asphalt binder, can play a significant role in the low-temperature cracking resistance. The addition of sulfur will form stronger molecular bonds with the asphalt molecules to form a three-dimensional lattice structure, thus enhancing the viscosity and storage stability of the modified asphalt [6]. Rubber processing oil is added due to its rich lightweight component, which can promote the swelling of SBS copolymers to Improve the low temperature crack resistance of SBS modified asphalt [5]. Besides, SBS copolymers alter the microstructure and composition of the asphalt, leading to improved low-temperature properties [5,6,7]. Therefore, it is imperative to study the influence of SBS content, sulfur, and rubber processing oil on the low-temperature properties of SBS-modified asphalt.

Compared with conventional asphalt, the low-temperature properties of SBS-modified asphalt consist of two parts: base asphalt deformation and SBS modifier deformation. It has been shown that polybutadiene (PB) with low glass transition temperatures (T_g_) could improve the rheological properties of the base asphalt at low temperatures [8]. However, the stiffness and m-value obtained from bending beam rheometer (BBR) test demonstrate that the low-temperature deformability of modified asphalt would be weakened if the SBS amount exceeds a particular range. Cao et al. [9] defined the temperature corresponding to the phase angle and loss of peak modulus value as the binder glass transition temperature (T_g_). Considering T_g_ as the evaluation parameter, the low-temperature performance of the modified asphalt would be improved with the increasing of SBS amount. Sassan et al. [10] investigated the low-temperature properties of SBS-modified asphalt by the rheological method. It was found that the rheological models were not consistent with different indexes for evaluating the low-temperature properties. Based on these findings, the addition of an SBS modifier, which could improve the low-temperature properties of base asphalt, was considered. However, the influence of SBS content on the low-temperature properties of SBS-modified asphalt is inadequate due to a variety of test methods and evaluation indicators. The polarity, molecular weight, and solubility parameters of SBS are much different from the asphaltenes, resulting in poor compatibility and stability among SBS and base asphalt. Dong et al. (2014) [2] elucidated the SBS with high aromatic content is more compatible with asphalt from the perspective of dispersion. J-F. Masson et al. (2005) [8] elucidated that asphalt with high light maltenes content would have a better miscibility with SBS copolymer. Sun et al. (2006) [11] found that sulfur addition formed a cross-linked network structure with asphalt, thus improved the storage stability of modified asphalt. Liang (2017) [7] discovered that the viscous characteristics decrease with the increase of polymerized sulfur content through the frequency sweep tests. The researches on rubber processing oil and sulfur have primarily focused on promoting the stability between base asphalt and SBS copolymers. Still, there is little research on its low-temperature performance, while the influence of different amounts remain sparse. 

From the existing research results, it can be seen that the rules to assess the influence of SBS content, sulfur, and rubber processing oil on the low-temperature performance of base asphalt are inconsistent due to the different evaluation indicators. Therefore, it is necessary to determine reasonable evaluation indicators before exploring the influencing factors of low-temperature properties of SBS-modified asphalt. Traditionally, low-temperature properties of the asphalt binders are tested using low-temperature penetration, low-temperature ductility, and Fraas breaking point test. Recent researches have confirmed that the conventional test methods cannot predict the cracking temperature accurately [12,13]. Meanwhile, contemporary testing procedures including the rheological tests and the correlated rheological data, like complex shear modulus (G*), phase angle (δ), creep stiffness (S), and creep rate (m), were employed to characterize the low-temperature performances of the asphalt binder [14,15,16,17,18]. This research aims to investigate the low-temperature properties of the SBS-modified asphalt with different amounts of SBS, sulfur, and rubber processing oil. Therefore, the orthogonal tests, which can replace the total tests and analyze the comprehensive influence of multiple factors with fewer tests, were used to design L3*3 tests in three different quantities. After that, the creep stiffness(S), creep stiffness rate (m), G-R constant, maximum force (Fmax), stretched elongation, and other indices obtained from the BBR experiment, DSR frequency sweeping test, and force-ductility test were conducted. In addition, a more reasonable index was determined to fairly evaluate the low-temperature performance of SBS-modified asphalt.

## 2. Materials and Methodology

### 2.1. Materials and Preparation

The base asphalt using SK-70 was provided by the SK Petroleum Asphalt Factory, South Korea. The conventional properties of the asphalt matrix, such as penetration, softening point, and ductility, have been given in Table 1. As the literature indicates, the structure type such as linear or star slightly influences the properties of modified asphalt. The SBS used in this research was come from the Yanshan Petrochemical Co., Ltd., Beijing, China, with contents ranging from 2% to 6% [19]. Table 2 presents its fundamental physical performance indices. Sulfur, which was selected as a cross-linking agent to improve the storage stability of SBS-modified asphalt with a content of 0%, 0.15%, and 0.3% respectively [5], came from Beijing Tiansuo Trading Company, and its main components have been given in Table 3. The rubber processing oil produced by Jiangsu Zhonghong Petroleum Asphalt Factory, China, was used to promote swelling of the modifier. Its content ranged from 0% to 4% [5]. Table 4 displays the details of the orthogonal test. It should be noted that different types of SBS-modified asphalt used in this research are termed XL-XS-XR for the convenience of the following charts. For example, the 2L-0.15S-2R contains 2% weight of SBS, 0.15% weight of sulfur, and 2% weight of rubber processing oil. In the Orthogonal experiment, the A, B, C represented the SBS, sulfur and rubber processing oil respectively, the Ⅰ, Ⅱ, Ⅲ represented three degrade of additive content respectively.

**Figure 1:**
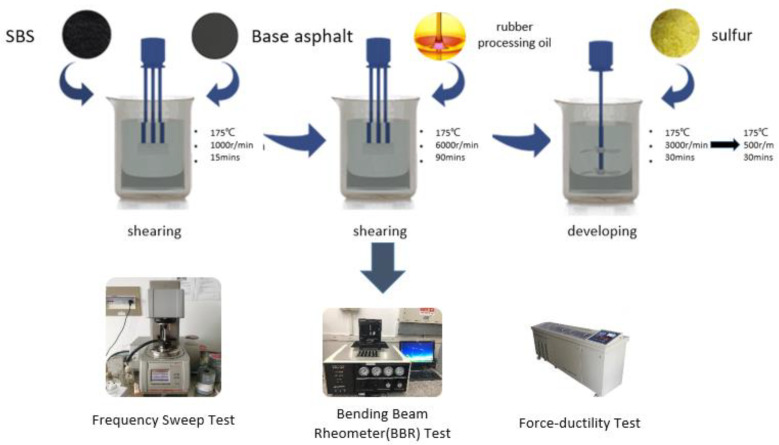
The flow chart of the research.

The experiments were conducted in the laboratory of transportation College, Southeast University. A uniform procedure was adopted to prepare the samples listed in Table 1d to minimize the influence of the process on the asphalt [5]. First, SBS was added to the base asphalt and stirred for 15 min under 1000 rpm rotation speed at 175 °C. Then, the corresponding amount of rubber processing oil were added to the mixture and sheared for 90 min under 6000 rpm rotation speed at 175 °C by a mechanical stirrer. Sulfur was then added to the mixture and stirred for another 30 min under 3000 rpm rotation speed. At last, the mixture were stirred for 30 minutes at a speed of 500 rpm to ensure the complete blending of SBS-modified asphalt. Once all samples are prepared, they should be used directly to prevent phase separation. The flow chart is shown in Figure 1.

### 2.2. Test Mothodology

#### 2.2.1. Frequency Sweep Test

An Anton Paar SmartPave 102 dynamic shear rheometer was used for this test at temperatures between 15 °C to 75 °C in interval of 15 °C to obtain the complex shear modulus (G*) and the phase angle (*δ*) at 0.1% strain. The test frequency is form 0.1 rad to 100 rads. The tests were performed between 15 °C and 30 °C with 8 mm diameter and 2 mm gap, while the others were undertaken with a 25 mm diameter and 1 mm gap geometry. The time-temperature superposition principle and the 2S2P1D model were also used to produce the master curve at a reference temperature of 15 °C [25,26]. The 2S2P1D model is a generalized model derived from the Huet-Sayegh model, consisting of two springs, two parabolic creep elements and a viscous pot, which can accurately describe the rheological properties of adhesive and asphalt mixtures. According to the established rheological curves, the storage and loss moduli under the condition of 0.005 rad/s can be calculated based on Equations (1) and (2). Finally, the G-R constant is obtained from Equation (3). The 2S2P1D model can be classified into seven parameters, and the expression of |$G^{*}$| is shown in Equation (4).

(1) $$\mathrm{lg}\omega_{r}=\mathrm{lg}\omega +\mathrm{lg}\alpha \left(T\right)$$

(2) $$\mathrm{lg}\alpha \left(T\right)=\frac{C_{1}\left(T-T_{r}\right)}{C_{2}+\left(T-T_{r}\right)}$$

(3) $$G-R\;\mathrm{Parameter}=G^{*}\times {\left(\cos\delta \right)}^{2}/\left(\sin\delta \right)\;(15\;{}^{\circ}C,\;0.005\;\mathrm{rad}/s)$$

(4) $$G^{*}\left(\omega \right)=G_{0}+\frac{G_{g}-G_{0}}{1+\mu {\left(i\omega \tau \right)}^{-k}+{\left(i\omega \tau \right)}^{-h}+{\left(i\omega \beta \tau \right)}^{-1}}$$

where, ‘$T_{r}$’ is the reference temperature, ‘T’ is the test temperature, ‘$C_{1}$′ and ‘$C_{2}$′ are the regression coefficients dependent on the type of asphalt binder, ‘$\omega_{r}$’ is the reduced frequency, ‘ω’ is the actual angular frequency, ‘$G^{*}$’ and ‘δ’are complex shear modulus and phase angle, respectively. ‘μ’ is a correction index, ‘ β’ is related to viscosity, ‘k’ and ‘h’ correlate with the material properties (0 < k < h < 1). When ω→0, ‘$G_{0}$′ is a static modulus. When ω→∞, ‘$\;G_{g}$’ is a glassy modulus.

#### 2.2.2. Bending Beam Rheometer (BBR) Test

According to ASTM D6648 [27], the BBR instrument (Cannon 9732-V31) can be used to determine the creep stiffness (S) and creep rate (m) of SBS modified asphalt. In this research, the stiffness and m-value were obtained under the temperature of −12 °C, −18 °C, and −24 °C, respectively. The critical PG low-temperature of asphalt is determined by the interpolation method. The critical low-temperature grade determined by stiffness (s) is obtained through logarithmic linear interpolation. The critical low-temperature grade determined by creep rate (m) is obtained through linear interpolation. The low-temperature properties of SBS modified asphalt were evaluated with $\Delta T_{c}$(Equation (5)) [28,29].

(5) $$\Delta T_{c}=T_{c\left(\mathrm{stiffness}\right)}-T_{c\left(m-\mathrm{slope}\right)}\;T_{c\left(\mathrm{stiffness}\right)}=T_{1}+\left(\frac{(T_{1}-T_{2})\cdot \left(log300-logS_{1}\right)}{logS_{1}-logS_{2}}\right)-10\;T_{c\left(m-\mathrm{slope}\right)}=T_{1}+\left(\frac{(T_{1}-T_{2})\cdot \left(0.3-m_{1}\right)}{m_{1}-m_{2}}\right)-10$$

where ‘$T_{1}$’, ‘$T_{2}$’, are the test temperature (°C) and ‘$S_{1}$’, ‘$S_{2}$’, ‘$m_{1}$’, ‘$m_{2}$’ are the corresponding test results of the BBR test, ‘$T_{c\left(\mathrm{stiffness}\right)}$’ (°C)is the critical temperature corresponding to the stiffness (s) is 300 MPa, and ‘$T_{c\left(m-\mathrm{slope}\right)}$’ (°C)is the critical temperature corresponding to the creep rate (m) is 0.3.

#### 2.2.3. Force-Ductility Test

The force-ductility test was conducted in the ductility tester (Hebei Tuofeng Instrument LYY-7F). An 8-shaped standard durability test model was selected with a loading speed of 5 cm/min at 5 °C to investigate the cohesive tensile performance [30]. Before the peak range of tensile deformation was reached, one tensile force every 1 mm was collected; afterward, one tensile force every 5 cm was collected. By calculating the area enclosed by the force-ductility curve, the viscosity and stiffness (which are related to the low-temperature properties of modified asphalt) were estimated.

## 3. Results and Discussion

### 3.1. G-R Parameter Analysis

According to the definition of the G-R parameter given above, this parameter corresponds to a very low frequency. In this test, the 2S2P1D model was conducted to deduce the complex modulus and phase angle at 15 °C and 0.005 rad/s.

The model calibration was accomplished by utilizing the Global Optimization in MATLAB software, and the model parameters are shown in Table 5. The reference temperature was 15 °C.

**Figure 2:**
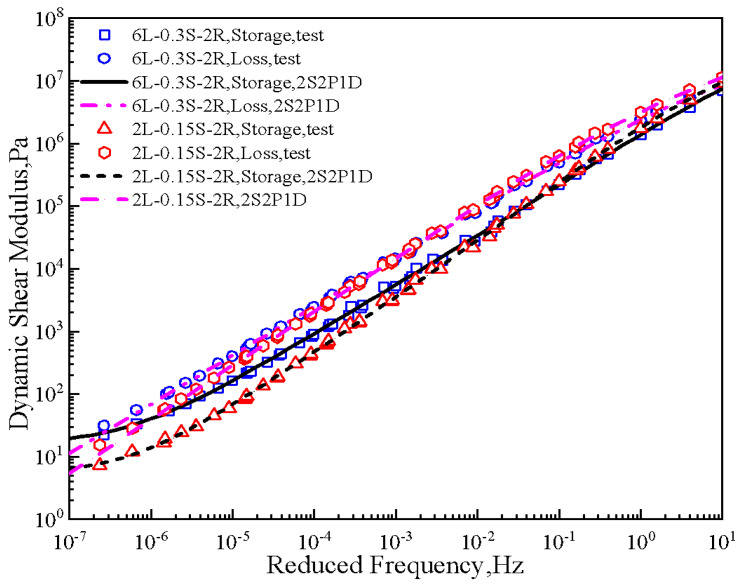
Modulus master curves of 6L-0.3S-2R and 2L-0.15S-2R samples.

**Figure 3:**
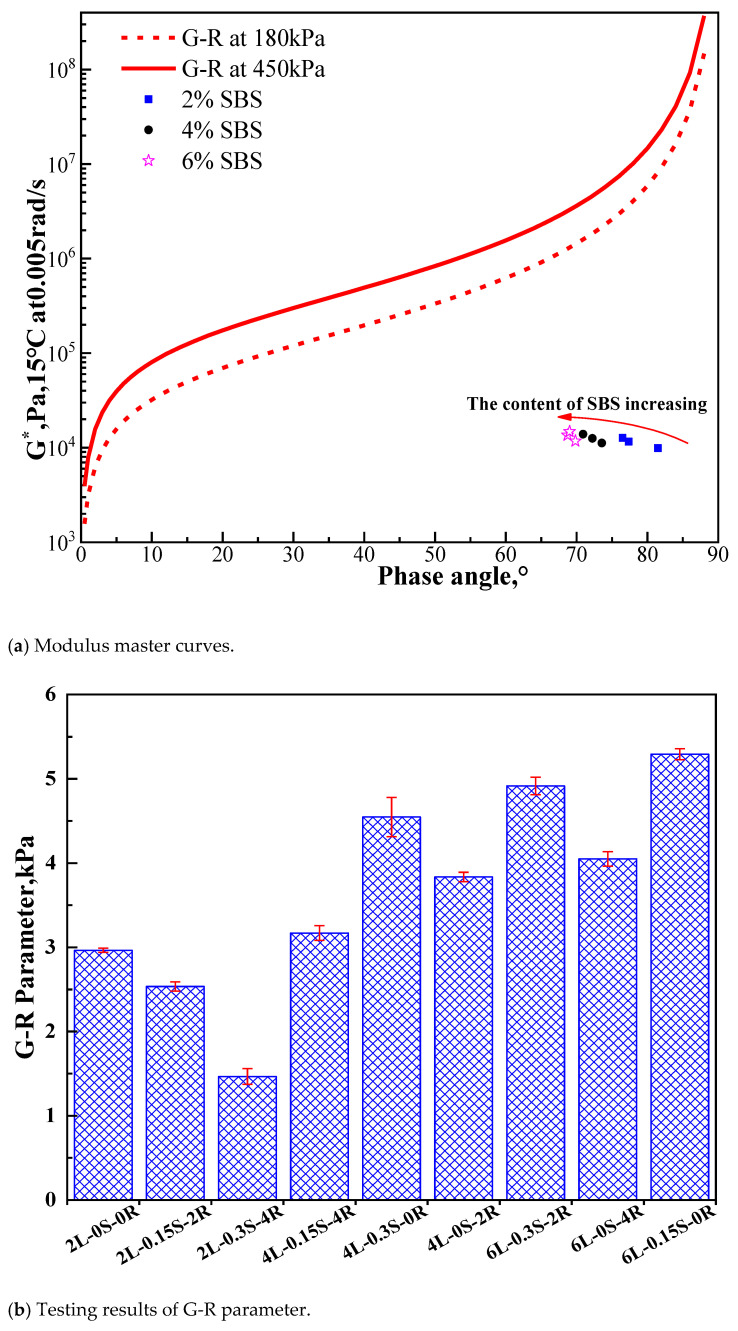
Modulus master curves and testing results of G-R parameter.

Storage modulus and loss modulus master curves for 6L-0.3S-2R and 2L-0.15S-2R samples are shown in Figure 2. The results implied a good fitting degree between the 2S2P1D model and the test data. In addition, the difference of storage modulus between the two samples at low and high frequencies was more evident than that of loss modulus. This difference may be attributed to the temperature sensitivity of the SBS polymer, which is lower than asphalt, especially at 6% content. Based on the storage and loss modulus master curves, the complex shear modulus and phase angle at 15 °C and 0.005 rad/s were calculated. Following this procedure, the G-R parameter shown in Figure 3a was determined and plotted on the black space diagram. Each point in the black space diagram represents the low-temperature properties of asphalt. The G-R parameter that exceeds 180 kPa indicates a risk of cracking in asphalt pavement, and the pavement will have severe block cracks and reflective cracks if the G-R parameter exceeds 450 kPa [31]. Therefore, with the increase of the G-R parameter, asphalt will become brittle and prone to cracking.

It can be seen from Figure 3a that the phase angle decreases sharply with the increase of SBS content, indicating that the elastic part of the asphalt binder increases along with the increase of SBS content. However, the complex modulus remains stable, which may be attributed to the similarity of modulus between SBS and asphalt at this frequency and temperature.

Figure 3b demonstrates the G-R parameter value of SBS modified asphalt under various combinations of additives. The G-R parameter increases, and the increase of SBS content mainly comes from the phase angle(δ). Figure 3b shows that the phase angle(δ) significantly influenced the G-R parameter value. Comparing the influence of rubber processing oil and sulfur with the same amount of SBS, it can be found that adding 4% rubber processing oil can significantly reduce the G-R parameter while adding 0.3% sulfur had no apparent effect. To some extent, rubber processing oil, which was rich in aromatic components that could soften the asphalt, positively affected the base asphalt. Further, the effect of sulfur on the SBS-modified asphalt is similar to rubber vulcanization which could generate a three-dimensional network structure to improve the complex modulus of cementing agent and reduce the phase angle in the process of modifying asphalt. However, this effect was not much pronounced in low frequency and had little influence on the G-R constant.

The results above illustrated that rubber processing oil could significantly improve the low-temperature properties of the SBS-modified asphalt, while sulfur barely influenced it. Besides, the increase of SBS content was negative to the low-temperature performance.

### 3.2. Analysis of BBR Test

**Figure 4:**
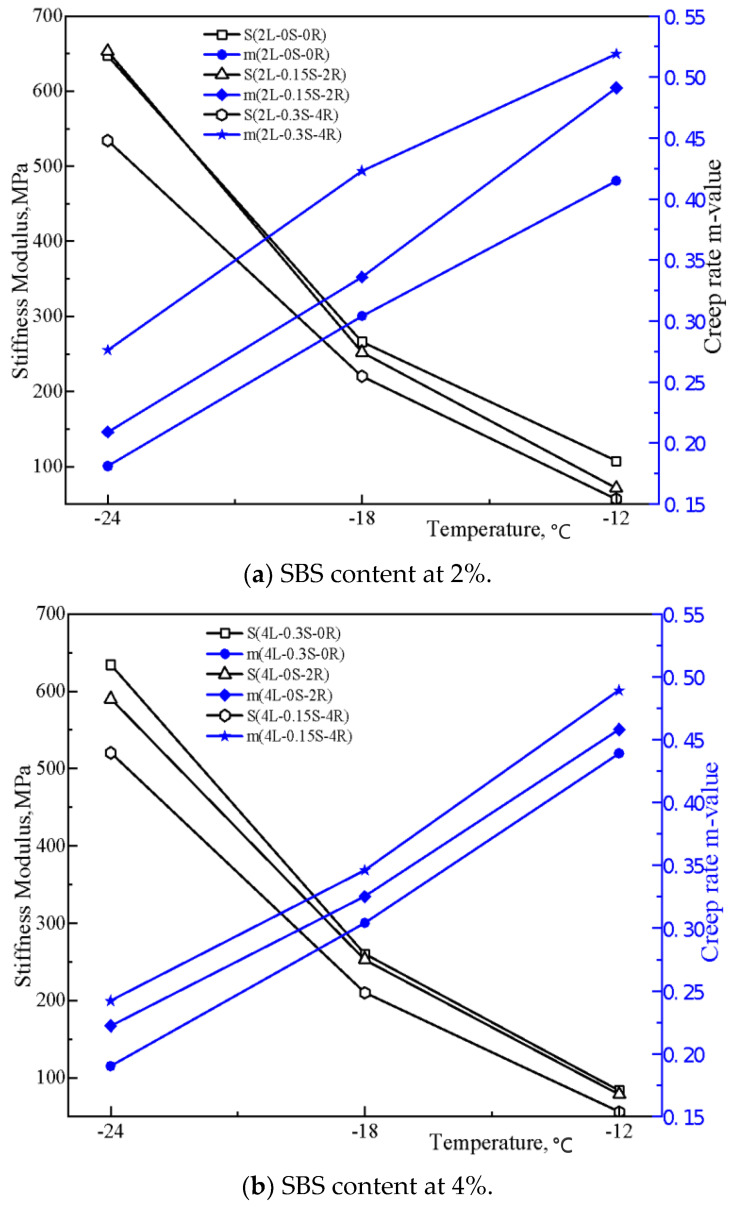

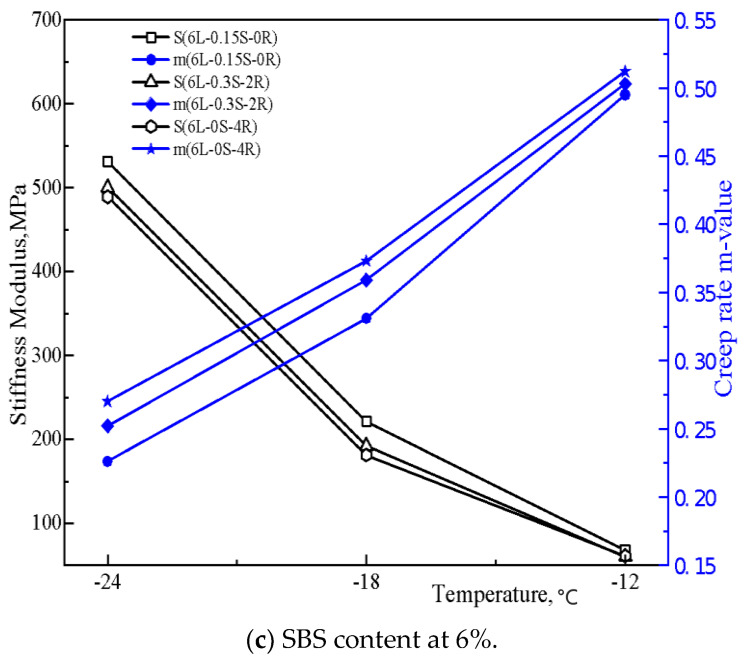
Test results of BBR.

The BBR test results have been assessed in Figure 4 by the stiffness and m-value at different testing temperatures. It could be seen that with an increase of SBS content, the stiffness values decrease relatively gently. When rubber processing oil was added to the base asphalt, the stiffness decreased, and the m-value increased sharply, especially with 4% rubber processing oil content. It was observed that the effect of rubber processing oil on the creep ability of asphalt binder is related to the SBS content. This phenomenon may originate from the absorption of the aromatic components during modification. When the SBS content is low, rubber processing oil will not be absorbed entirely to supply surplus aromatic components to the asphalt, resulting in a better low-temperature performance of SBS-modified asphalt. However, the influence of sulfur under different contents is not obvious and will be further understood through orthogonal test analyses.

PG low temperature of BBR test results are shown in Table 6. Since the PG classification rule in SHRP fails to accurately classify the low-temperature degree of SBS-modified asphalt in this test, continuous classification methods have been adopted. It can be found that the low-temperature grade of SBS-modified asphalt decreases with the increase of SBS and rubber processing oil, but this influence was not significant.

**Figure 5:**
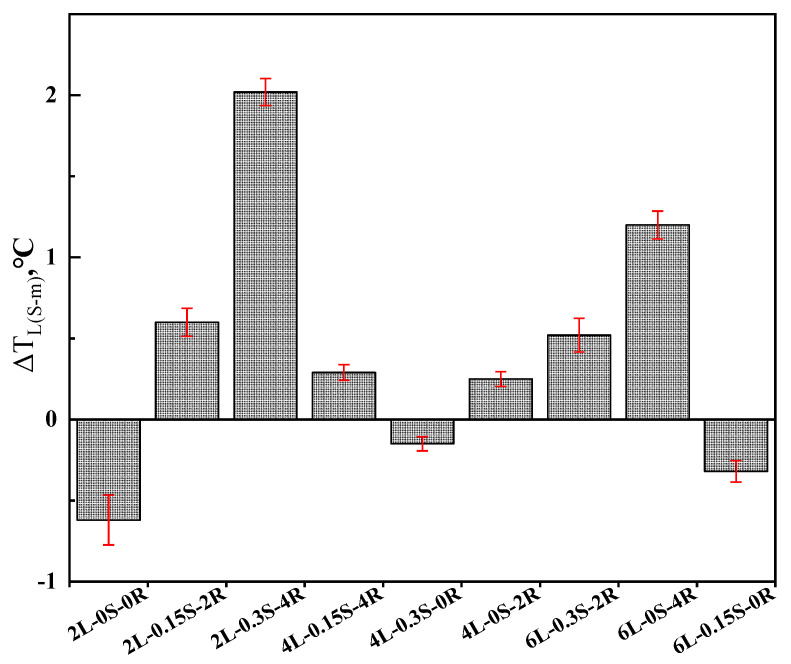
Δ*T_c_* of SBS-modified asphalt.

However, these indexes only consider the stiffness and m-value, whose calculations are one-sided. Therefore, Δ*T_c_* is adopted to better characterize the factors affecting the low-temperature properties of SBS-modified asphalt. According to the research results of Anderson et al. (2011) [28], Δ*T_c_*, the difference between critical PG low temperature determined by ‘S’ and ‘m’ values, has a good correlation with cracks. Statistics verified that the critical value of Δ*T_c_* is −2.5 °C; the smaller the Δ*T_c_* value is, the more brittle the asphalt is and the more likely it is to crack. As illustrated in Figure 5, Δ*T_c_* of modified asphalt is all below 0 when SBS content is 2%. It implies that the modified asphalt is controlled by m-value and is easy to crack at this instant. When increasing the rubber processing oil content to 4%, ‘Δ*T_c_*’ changes from negative to positive, especially at 2% SBS content. It can also be explained that the residual rubber processing oil is rich in aromatic components, which can promote the flowability of asphalt binders at low temperatures.

Comparing the ‘Δ*T_c_*’of samples 2L-0S-0R, 4L-0.3S-0R and 6L-0.15S-0R, it can be identified that sulfur addition can make the Δ*T_c_* approach to zero, which implies that sulfur can balance out the stiffness modulus and creep rate. In addition, Δ*T_c_* of 4L-0.3S-0R sample is minimum, contributing to the reasonable amount of SBS and sulfur and resulting in a three-dimensional network structure in the modified asphalt system. The formation of the three-dimensional network system will enhance the intermolecular interaction force, thereby reducing the creep deformation rate.

### 3.3. Analysis on Force-Ductility

**Figure 6:**
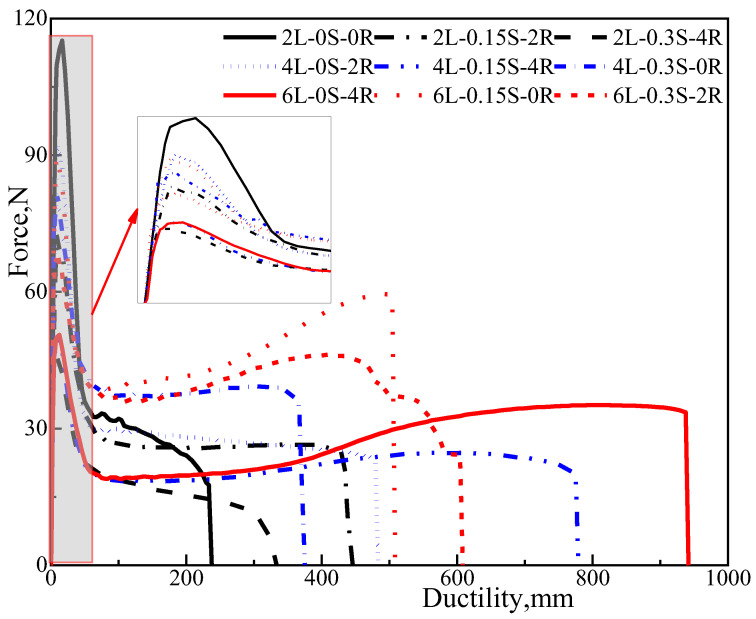
Force-ductility curves for nine different types of polymer modified bitumen.

The F_max_ and stretched elongation for various types of modified asphalt obtained from the force-ductility test are shown in Figure 6. As seen from the results, no apparent relationship could be ascertained between SBS or sulfur content with F_max_. However, the maximum force tends to decrease significantly with the increase of rubber processing oil content. It was also observed that the force-ductility test is mainly determined by the composition of the asphalt matrix, while the good compatibility between asphalt and rubber processing oil changes the composition of the asphalt matrix directly. The increase of aromatic components reduces the bonding force of molecules, resulting in the decrease of F_max_.

Additionally, by evaluating the effect of rubber processing oil with 4% SBS on the reduction of maximum force-ductility, it can be concluded that F_max_ will reduce slightly along with the increase of SBS content. As discussed earlier, it may be attributed to the absorption of aromatic components, which can relieve the effect of F_max_. Besides, the tensile force and fracture length of modified asphalt increase sharply with increasing SBS content in the late tensile phase, which implies the toughness enhancement of the modified asphalt.

Comparing the addition of rubber processing oil and sulfur at the same SBS content reveals that sulfur impacts the second stage tensile force, whereas the rubber processing oil affects the fracture length. The reasons for such behaviors are that sulfur addition can built a three-dimensional network, and the rubber processing oil addition can provide an aromatic component to asphalt. Overall, the increase of SBS content can improve the low-temperature tensile properties of asphalt and can resist cracking in a more extensive range.

**Figure 7:**
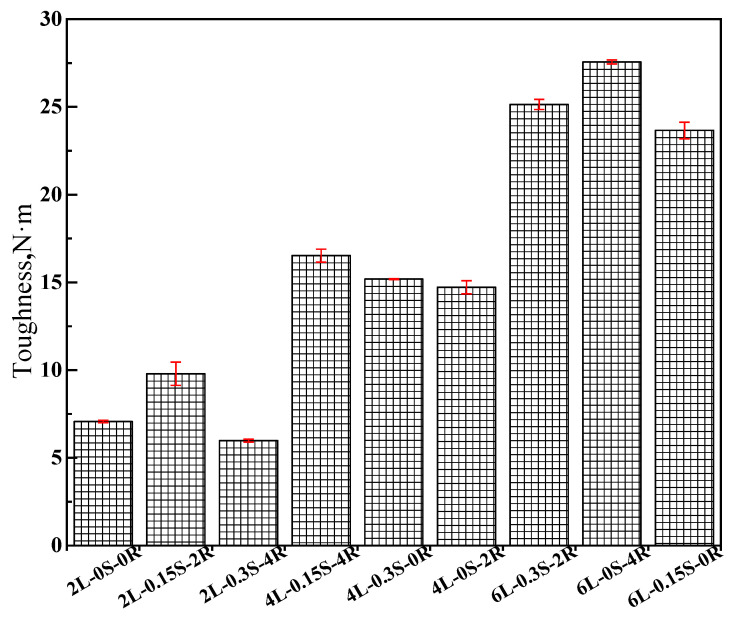
Toughness of all tested binders.

According to Sun’s force-ductility research on modified asphalt [32], Figure 7 illustrates the area surrounded by the ductility curve of samples to quantify the increased ratio of the toughness of SBS-modified asphalt. The results indicate that the SBS increase can strengthen the toughness of modified asphalt, and the sulfur and rubber processing oil have a certain role in it. Excessive rubber processing oil will harm the low-temperature crack resistance, and as such, it is not recommended for low SBS content.

### 3.4. Sequencing Analysis of Low-Temperature Parameter Based on Orthogonal Test

The experimental data indicate that certain efforts were taken after adding SBS, sulfur, and rubber processing oil, while the results obtained by the G-R parameter are contrary to those of Δ*T_c_* and the toughness. For example, the SBS increase is negative to the low-temperature properties of modified asphalt, whereas the results of other tests are beneficial. It may be attributed to establishing the G-R parameter, which is mainly based on the number of fractures. The G-R parameters of all modified asphalts in this study are far less than the critical cracking value. Therefore, G-R parameter is more suitable to evaluate the low-temperature cracking resistance of aging asphalt. Follow-up orthogonal tests are to be conducted only for Δ*T_c_* and toughness. The orthogonal test results of the multi-factor and single index are to be analyzed by the visual analysis method. Table 7 is the result of the orthogonal tests, in which K_1_, K_2_, and K_3_ are the sum of the indexes below the level I, II, and III, respectively. The range represents the magnitude of the influence of factors on the respective indices. According to Table 5, when Δ*T_c_* is taken as the index, it can be found that the range of SBS content is the largest, followed by rubber processing oil and finally sulfur. Although there is not a very high degree of disparity in these factors, it can still be deduced that the influence of each factor on the low-temperature performance is SBS > rubber processing oil > sulfur. When the evaluation index is toughness, the order is the same, but the range of SBS content is greater than that of sulfur and rubber processing oil. The SBS content has a more significant influence on the low-temperature properties since it can resist the generation and further development of cracks. Meanwhile, the rubber processing oil can delay the generation of the cracks, which can obviously enhance the low-temperature performance of SBS-modified asphalt.

## 4. Conclusions and Recommendations

In this paper, the effects of SBS content, sulfur, and rubber processing oil on the low-temperature performance of the SBS-modified bitumen were studied based on the orthogonal test. The low-temperature performance was characterized by the frequency sweep test, BBR test, and force-ductility test. The conclusions drawn from the experimental results are as follows.

(1) According to the results of the BBR, force-ductility and G-R test, addition of rubber processing oil can supplement the loss of maltene and improve the low-temperature properties significantly. However, excessive amounts of rubber oil was detrimental to adhesive toughness of SBS modified asphalt.

(2) The BBR test illustrates that low-temperature performance of SBS-modified asphalt is limited by the lack of m-value. The addition of sulfur and rubber processing oil improves its m-value and promotes a balance between stiffness and m-value in SBS-modified asphalt.

(3) According to the direct analysis of the orthogonal test, the influence degree of low-temperature properties of SBS-modified asphalt was SBS > rubber processing oil > sulfur.

Only SBS-modified asphalt binders in long-term aging condition based on one kinds of base asphalt were examined in this study. Additional aging condition, different compositions of base asphalt and asphalt mixtures should be studied in the future to verify the conclusions of this study.

## Figures and Tables

**Table 1:** Main performance indexes of base asphalt.

Physical Properties	Units	SK-70#	Test Method
Penetration (25 °C, 100 g, 5 s)	0.1 mm	67.6	ASTM D5 [20]
Softening point (R&B)	°C	48.6	ASTM D36 [21]
Ductility (15 °C, 5 cm/min)	cm	>100	ASTM D113 [22]
Density	g/cm^3^	1.02	ASTM D792 [23]
PG degrade	-	PG64	ASTM M320 [24]

**Table 2:** SBS basic physical performance indexes.

Structure Type	Styrene-Butadiene Ratio	Breaking Elongation (%)	Tensile Strength (MPa)	Melt Flow Rate (g·10·min^−1^)
Linear	30/70	800	26	0.5

**Table 3:** Main components and content of sulfur.

Name	Content (%)
Rhombic sulfur	95.71
NH_4_SCN	1.33
(NH)_4_S_2_0_3_	1.13
Others	1.83

**Table 4:** Orthogonal experimental schemes.

Number	Factor A (SBS/%)	Factor B (Sulfur/%)	Factor C (Rubber Processing Oil/%)	Program	Name
1	2	0.00	0	AⅠBⅠCⅠ	2L-0S-0R
2	2	0.15	2	AⅠCⅡDⅡ	2L-0.15S-2R
3	2	0.30	4	AⅠBⅢCⅢ	2L-0.3S-4R
4	4	0.15	4	AⅡBⅡCⅢ	4L-0.15S-4R
5	4	0.30	0	AⅡBⅢCⅠ	4L-0.3S-0R
6	4	0.00	2	AⅡBⅠCⅡ	4L-0S-2R
7	6	0.30	2	AⅢBⅢCⅡ	6L-0.3S-2R
8	6	0.00	4	AⅢBⅠCⅢ	6L-0S-4R
9	6	0.15	0	AⅢBⅡCⅠ	6L-0.15S-0R

**Table 5:** The model parameters of all tested binders.

Type	$G_{e}(\mathrm{MPa})$	$G_{g}\;(\mathrm{MPa})$	μ	k	h	β	$\tau_{0}/10^{-3}$	$C_{1}$	$C_{2}$
2L-0S-0R	0	1420.31	13.26	0.384	0.825	9523.4	0.24	11.846	82.423
2L-0.15S-2R	0	1120.31	10.26	0.414	0.832	8921.9	0.16	11.529	83.295
2L-0.3S-4R	0	866.776	8.632	0.426	0.862	7845.3	0.13	9.824	80.477
4L-0.15S-4R	0	1330.74	9.458	0.389	0.831	10,000.5	0.53	10.992	78.354
4L-0.3S-0R	0	1715.96	9.593	0.351	0.795	11,964.9	0.61	11.924	81.652
4L-0S-2R	0	1545.91	9.135	0.374	0.805	10,974.6	0.69	11.433	80.299
6L-0.3S-2R	0	1499.46	10.94	0.316	0.750	12,286.9	0.56	11.302	81.853
6L-0S-4R	0	1290.49	9.424	0.320	0.790	11,095.2	0.79	12.449	102.402
6L-0.15S-0R	0	1799.87	13.57	0.315	0.775	13,174.2	0.36	12.839	95.286

**Table 6:** PG Low temperature of BBR test for all tested binders.

Binder Type	T_L,m_	T_L,S_	T_L_	ΔT_L (S-m)_
2L-0S-0R	−28.19	−28.81	−28.19	−0.62
2L-0.15S-2R	−29.70	−29.10	−29.10	0.60
2L-0.3S-4R	−33.02	−30.10	−30.10	2.92
4L-0.15S-4R	−30.65	−30.36	−30.36	0.29
4L-0.3S-0R	−28.21	−28.36	−28.21	−0.15
4L-0S-2R	−29.45	−29.20	−29.20	0.25
6L-0.3S-2R	−31.31	−30.79	−30.86	0.52
6L-0S-4R	−32.25	−31.05	−30.98	1.20
6L-0.15S-0R	−29.77	−30.09	−29.84	−0.32

**Table 7:** Orthogonal test results.

Indexes		A (SBS)	B (Sulfur)	C (Rubber Oil)
|Δ*T_c_*|	K_1_	4.140	2.070	1.090
	K_2_	0.690	1.210	1.370
	K_3_	2.040	3.590	4.410
	Range R	3.450	2.380	3.320
	Primary and secondary factors	A > C > B		
Toughness	K_1_	22.843	49.353	45.913
	K_2_	46.426	49.976	49.652
	K_3_	76.359	46.299	50.063
	Range R	53.516	3.677	4.150
	Primary and secondary factors	A > C > B		

## Data Availability

The data will be available on demand.
